# Point-of-Care Ultrasound Unveils Life-Threatening Conditions: A Case Series Highlighting Its Vital Diagnostic Role

**DOI:** 10.7759/cureus.47513

**Published:** 2023-10-23

**Authors:** Muhammad Ghallab, Salman Ashfaq, Nicole C Noff, Daniel Miller, Asma Hosna, Allison Foster, Karim Makhoul, Avish Parikh, Ricardo Lopez

**Affiliations:** 1 Internal Medicine, Icahn School of Medicine at Mount Sinai, NYC Health and Hospitals, Queens, New York, USA; 2 Critical Care Medicine, Icahn School of Medicine at Mount Sinai, NYC Health and Hospitals, Queens, New York, USA

**Keywords:** pocus echocardiogram, ventricular thrombus, type a aortic dissection, pulmonary critical care, point-of-care-ultrasound

## Abstract

Point-of-care ultrasound (POCUS) has emerged as a powerful diagnostic tool in healthcare, offering rapid and cost-effective evaluation of cardiovascular and respiratory conditions. This case series highlights the vital role of POCUS in diagnosing life-threatening conditions and emphasizes the need for adequate training in its use. The first case describes a patient with chest pain, where POCUS revealed findings suggestive of thoracic aortic dissection, leading to timely transfer and surgical repair. The second case involves a patient with altered mental status and hypoxia, where POCUS identified a right atrial thrombus leading to thrombectomy. The discussion explores the expanding applications of POCUS in various medical specialties, including critical care and trauma, and its potential to improve patient outcomes. While POCUS has shown great promise, it remains a user-dependent technology, necessitating comprehensive training and collaboration among healthcare professionals to ensure its effective and safe use.

## Introduction

Point-of-care ultrasound (POCUS) is described as the stethoscope of the future, allowing for rapid, cost-effective real-time evaluation of cardiovascular and respiratory pathology [1-3]. Rather than waiting for formal imaging studies, POCUS is a convenient portable handheld tool that facilitates an immediate investigation of differential diagnoses at the bedside and enhances the speed of clinical interpretation, and allows for sequential exams over time. Its use across hospital settings and within medical training programs has steadily grown over the past few decades and is available to all practitioners of all specialties [1]. According to a systematic review by the American College of Physicians, the rationale for adding POCUS to the standard diagnostic pathway encompasses several considerations. First, POCUS increased the proportion of correct diagnoses by 32% when used in addition to the standard diagnostic pathway. Second, the test accuracy, particularly sensitivity, of standard diagnostic testing with the addition of POCUS is better than the test accuracy of the standard diagnostic pathway alone without a substantial tradeoff in specificity. Third, it is unlikely that it is directly associated with serious harm, and finally, it is a cost-effective test [2]. Ultrasound is a user-dependent technology and requires adequate training [3]. Realizing the importance of proficiency in POCUS, the American College of Emergency Physicians recommends completing 150-300 POCUS exams during Emergency Medicine residency [1]. Here we present two cases, thoracic aortic dissection and right atrial thrombus, in which the use of POCUS was crucial in diagnosing life-threatening conditions in a timely manner.

## Case presentation

Case 1

**Figure 1:**
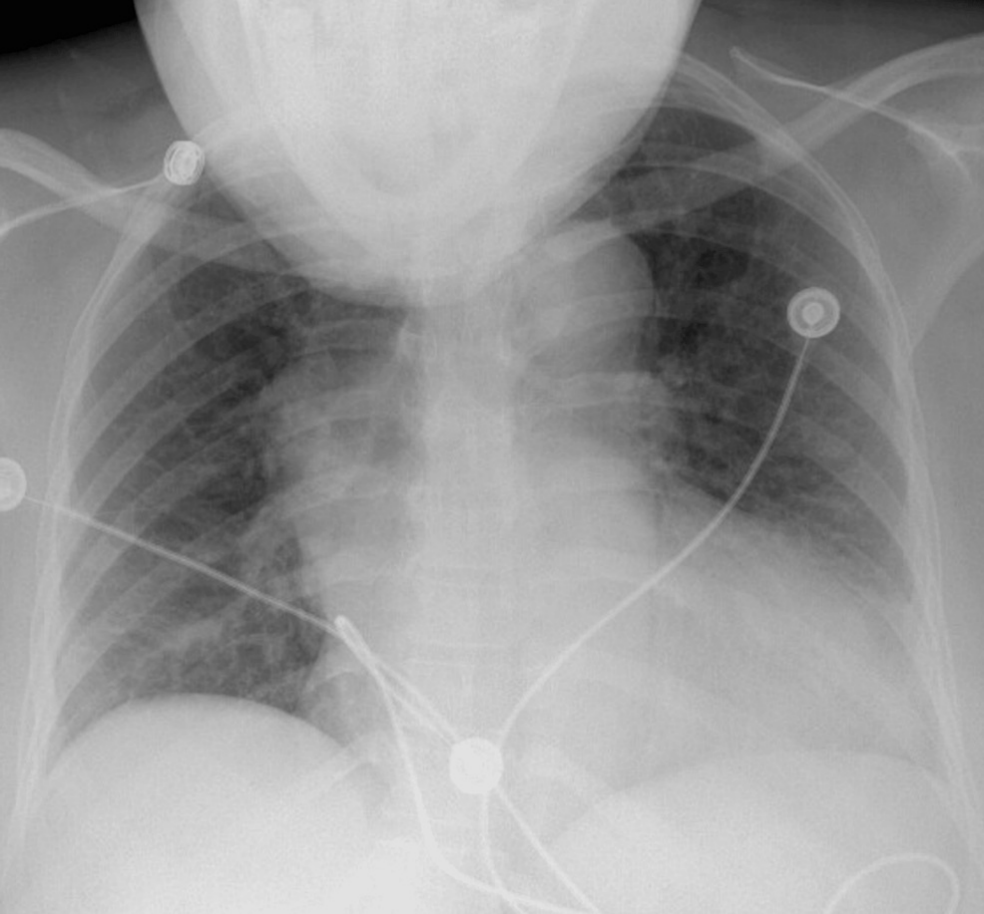
Case 1: Chest X-ray revealing wide mediastinum tortuous aorta

This is a case of a 49-year-old woman with a past medical history of hypertension who presented to our hospital with the chief complaint of mild chest pain manifesting on and off since the morning of the presentation. The patient expressed nonspecific mild sensations of chest pain in the past that has never been bothersome or required any medical intervention. During this presentation, the patient's pain had been migrating all over the precordium. The patient was normal in appearance and did not seem in acute distress. The patient was hemodynamically stable, not tachycardic or hypotensive. She did not have any murmurs on auscultation. Lung sounds were normal. She did not manifest any focal neurological deficit on physical examination. A chest X-ray revealed an enlarged cardiac silhouette, wide mediastinum, and tortuous aorta (Figure 1).

**Figure 2:**
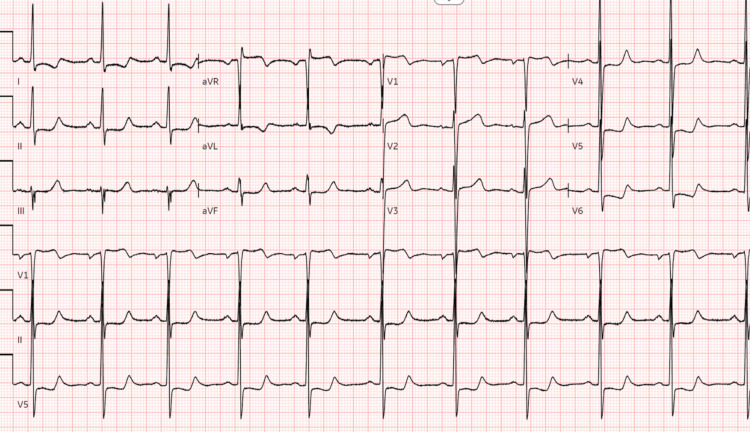
Case 1: Electrocardiogram revealing left ventricular hypertrophy.

EKG revealed findings consistent with left ventricular hypertrophy (Figure 2).

Serial troponin was negative. Pro-BNP was 876 pg/ml. D-dimer was 3058 ng/ml. Lactate on venous blood gas was 3.7 mmol/L. The patient was admitted for further evaluation and work-up. A point-of-care ultrasound (POCUS) was obtained for the evaluation of the heart and lungs. Cardiac POCUS revealed a dilated aortic root, a dissecting flap at the level of the ascending aorta at the parasternal long axis view (Videos 1-3), and a dissecting flap at the level of the abdominal aorta at the subcostal view.

**Video 1:** Case 1: Point-of-care cardiac ultrasound, parasternal long axis view revealing a dilated aortic root and dissecting flap at the level of the ascending aorta.

**Video 2:** Case 1: Point-of-care cardiac ultrasound, subcostal view (transverse axis) revealing a dissecting flap at the level of the abdominal aorta.

**Video 3:** Case 1: Point-of-care cardiac ultrasound, subcostal view (longitudinal axis) revealing a dissecting flap at the level of the abdominal aorta.

**Figure 3:**
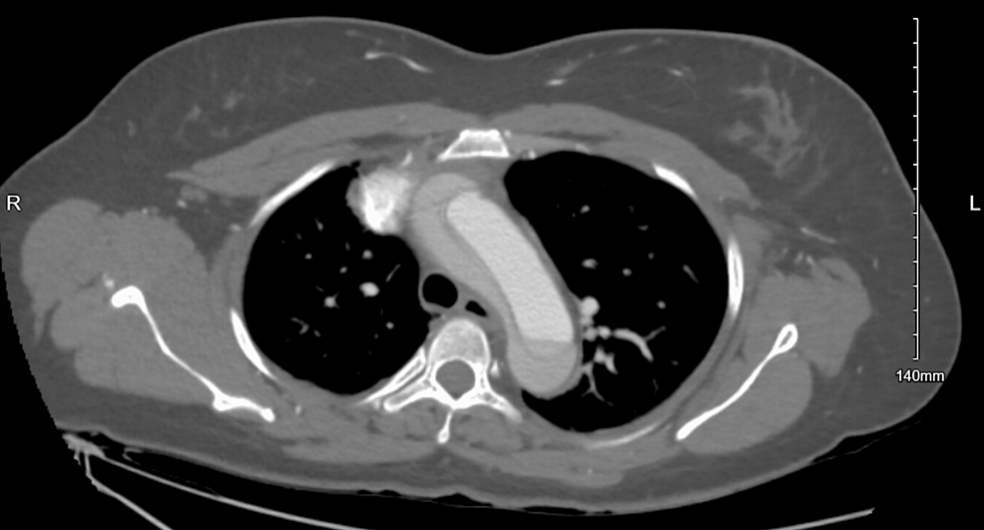
Case 1: Aortic dissection at the aortic arch

**Figure 4:**
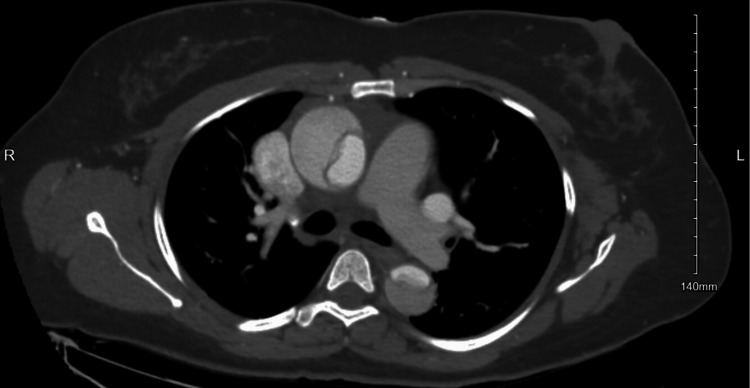
Case 1: Aortic dissection in the ascending and descending aorta

**Figure 5:**
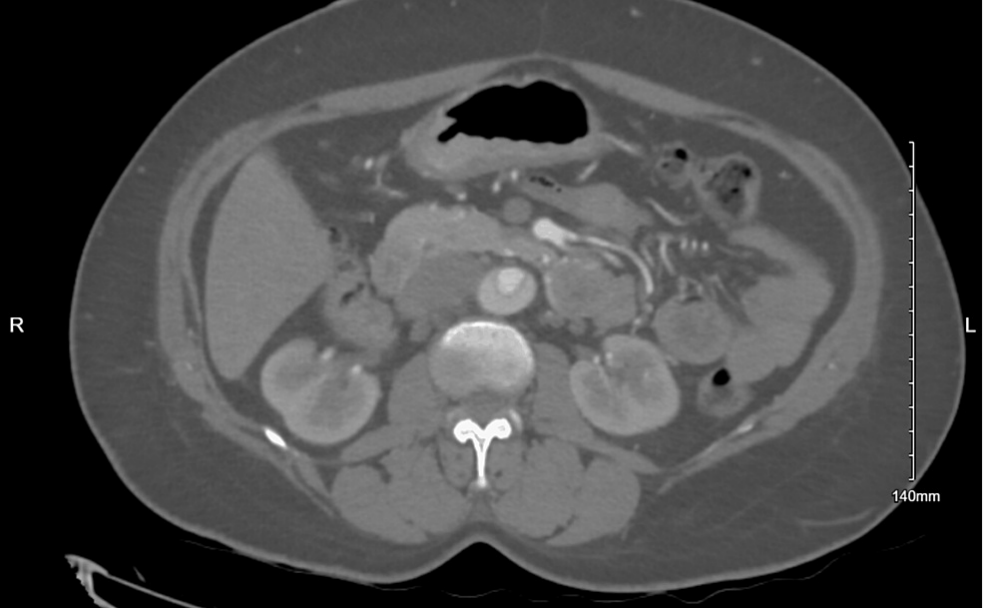
Case 1: Aortic dissection at the level of the lower abdominal aorta

CT angiography of the chest and abdomen was obtained and confirmed a 5.5 cm fusiform aortic aneurysm with Stanford type A / DeBakey type 1 thoracic aortic dissection; the dissection was found beginning at the level of the aortic valve and extends into the right common carotid artery superiorly and into the abdomen, terminating within the right external iliac artery inferiorly (Figures 3-5).

With a diagnosis of aortic dissection, IV esmolol was initiated for control of the blood pressure, and the patient was emergently transferred to a tertiary center and underwent emergent repair of the thoracic aortic dissection. The patient then achieved full recovery and was discharged after a few days.

Case 2

A 74-year-old female with a past medical history of dementia, with a recent history of right hemiarthroplasty secondary to a right femoral neck fracture two weeks earlier, presented to the emergency department with the complaint of decreased oral intake for a few days and altered mental status. She was recently discharged from the rehabilitation center. The patient was found to be drowsy and developed a rash after taking gabapentin for her hip pain. As per the family member, the patient had difficulty swallowing for a few days, associated with lethargy and exertional dyspnea. Her physical exam in the emergency department was significant for tachycardia, diffuse erythematous maculopapular rash, cold extremities with peripheral cyanosis, alert, oriented only to herself. A stroke protocol was initiated, and a computed tomography (CT) head was negative for acute hemorrhage or infarction. CT angiography head and neck did not reveal any dissection, pseudo aneurysm, or significant vascular abnormalities.

On admission, lab results were significant for acute kidney injury with a creatinine of 2.61 mg/dl, eGFR 19 ml/min/1.73 m^2^, lactate 6.4 mmol/L, troponin 0.592 ng/ml, pro-BNP 14110 pg/ml, and D-dimer 9305 ng/ml (Table 1).

**Table 1:** Case 2: Initial laboratory results of the patient on admission. ALT: Alanine transaminase, AST: Aspartate transaminase, ALP: Alkaline phosphatase, GFR: Glomerular filtration rate

Laboratory parameter	Reference range	Patient’s value
White blood cell	4.80-10.80 x 10^3^	10.91
Hemoglobin level	12-16 g/dL	13.86
Neutrophil %	44-70%	79.4%
Troponin	<0.010	0.592
Pro-BNP	1-125 pg/ml	1,4110
D-dimer	<370 ng/ml	9,305
Lactate	0.6-1.4 mmol/L	6.4
Sodium	136-145 mmol/L	156
Chloride	98-106 mmol/L	116
HCO3	22-29 mmol/L	19
Creatinine	0.50-1.20 mg/dl	2.61
Blood urea Nitrogen	6-23 mg/dl	44
Anion Gap	8-16 mEq/L	21
Glomerular filtration rate	>60 ml/min/1.73 m^2^	19
Creatine Kinase	20-170 U/L	348
Alkaline phosphatase	35-104 U/L	121
Aspartate transaminase	5-32 U/L	65

**Figure 6:**
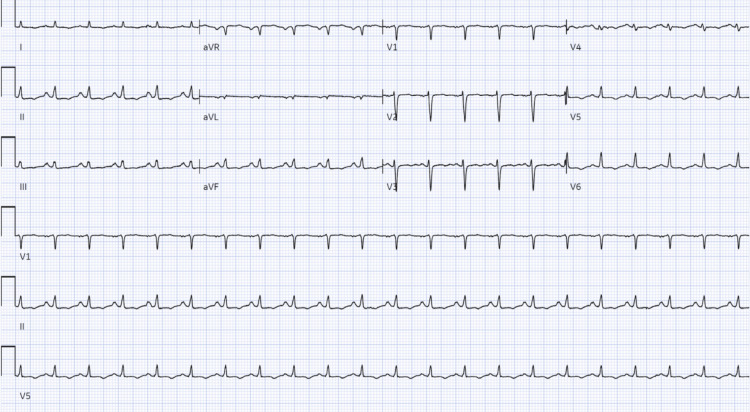
Case 2: Electrocardiogram revealing T wave inversions in inferolateral and low voltage in the limb leads.

EKG showed sinus tachycardia with nonspecific T wave Inversions in inferolateral leads and low-voltage limb leads (Figure 6).

On admission, the patient was found to be hypoxic and hypotensive. Bedside POCUS showed mildly decreased ejection fraction and dilated inferior vena cava. The patient was transferred to the intensive care unit. A right internal jugular triple-lumen catheter was placed, and norepinephrine was started. Cardiology service was consulted, and the patient was treated with aspirin 325 mg, ticagrelor 180 mg, and heparin infusion was started at a therapeutic dose for the treatment of non-ST segment elevation myocardial infarction (NSTEMI) type 1. Given that the patient had tachycardia, shortness of breath, recent surgical history, and elevated D-dimer, the probability of pulmonary embolism was high, but a computed tomographic angiogram of the chest could not be done due to worsening kidney function. The patient was unstable for the ventilation-perfusion scan. Troponin trended up from 0.592 ng/ml to 0.816 ng/ml. Bedside POCUS revealed a notable thrombus in the right atrium (Video 4), right-sided heart dilation, and non-compressible femoral veins in the bilateral lower extremity, suggesting deep vein thrombosis (DVT).

**Video 4:** Case 2: Right atrial thrombus visualized on point-of-care cardiac ultrasound

Heparin treatment was continued; the patient was started on antibiotics for possible septic shock and a warming blanket for hypothermia. Later in the day, the patient became more hypoxic, and her mental status deteriorated. She was then intubated. Massive pulmonary embolism was highly suspected, however given the history of recent surgery intravenous thrombolytic therapy was contraindicated, so the patient was transferred to a tertiary center for thrombectomy. After the thrombectomy, the patient continued to be managed for obstructive shock and cardiogenic shock, thought to be secondary to stress-induced cardiomyopathy. She was extubated eventually and transferred to the medicine floor for further management.

## Discussion

Point-of-care ultrasonography (POCUS) allows for quick, real-time evaluation of cardiovascular and pulmonary pathologies and ensures real-time monitoring of interventions. Over the past decade, interest and literature on the use of POCUS have increased exponentially [1]. This is especially true in the realm of critical care medicine, where the use of POCUS can reduce procedure risk and aid in the timely diagnosis of life-threatening pathologies and can thus lead to timely and early intervention [4]. POCUS has shown to be equally efficient in diagnosing abdominal aortic aneurysms and DVT when compared with formal ultrasonography and has also been shown to aid in diagnosing pneumonia and decompensated heart failure owing to its high accuracy.

POCUS evaluation expanded during the pandemic in evaluating patients with COVID-19. Extensive studies were performed on using POCUS in the evaluation of clinical outcomes in patients with severe COVID-19 disease. They showed good effectiveness when used with a multi-organ approach rather than lung ultrasound alone [5]. Some studies also suggested a correlation between interstitial pneumonia in COVID-19 patients and CO2 retention, with the evaluation of lung aeration scores (LAS) [6]. This speaks to the versatile nature of real-time POCUS evaluations, which gives clinicians a chance at early intervention.

POCUS is now being used in the realm of trauma as well, with POCUS showing high sensitivity and specificity in diagnosing life-threatening pneumothorax, hemothorax, and lung contusion [7]. Outside of trauma, POCUS has shown to be an invaluable tool in diagnosing medical and surgical emergencies such as aortic dissections and clots in transit, as discussed in our patients.

This, however, does not mean that POCUS does not have its limitations. The biggest limiting factor of POCUS is its being user-dependent. Many diagnoses can be missed, or misdiagnoses can be made if poor technique is applied during evaluation. Another factor is the comfort level of many providers in performing POCUS evaluations in the setting of a lack of formal training in the technique. The European Society of Radiology has released best practice recommendations and has suggested formal, continuous, and adequate training in ultrasound to provide quality examinations [4]. POCUS is also less sensitive in diagnosing certain medical conditions, such as acute pulmonary embolism when compared to the gold standard, which is CT imaging.

We demonstrate the use of POCUS in the evaluation and early interventions of life-threatening conditions by using best practice recommendations, including quality examination, the use of imaging documentation in a centralized system, and adequate hygiene measures. In our opinion, POCUS is an invaluable resource that is still underutilized when compared to its potential usage with a cost-effective point-of-care evaluation that can be performed serially with no additional risk to the patients. Formal training in the use of ultrasound is key in order to perform adequate evaluations, increase provider confidence, and reduce the chances of misdiagnosis when the evaluation is done. Collaboration with radiologists should also be considered during the formulation of training courses [4], as well as critical care providers in order to ensure good quality training.

## Conclusions

POCUS has proven to be a valuable tool in unveiling life-threatening conditions, with its everyday use rising to an all-time high during the COVID-19 pandemic. Not only is it efficient for evaluating cardiovascular and pulmonary pathologies, but it also allows for real-time monitoring of interventions. Here we presented two cases, thoracic aortic dissection, and right atrial thrombus, that accurately portrayed the crucial role that POCUS plays in the prompt assessment of patients. To best optimize its diagnostic value, formal training should be implemented for all health care professionals, not just emergency medicine, concomitantly with the collaboration of radiologists and critical care providers.
